# Eosinophilic lung disease with plastic bronchitis and bronchiectasis in an adolescent male

**DOI:** 10.1002/ccr3.1853

**Published:** 2018-10-26

**Authors:** Habiba Hussain, Nizar Kherallah

**Affiliations:** ^1^ University of Illinois College of Medicine at Peoria Peoria Illinois

**Keywords:** bronchiectasis, bronchoscopy, eosinophilic lung disease, interstitial lung disease, plastic/cast bronchitis

## Abstract

Any case of unresolving chronic pneumonia with expectoration of thick mucoid plugs should have high index of suspicion for plastic/cast bronchitis, requiring early flexible bronchoscopy with lavage and histopathologic evaluation. Associated presence of idiopathic chronic eosinophilic pneumonia is unusual and could be considered as a cause for atypical plastic bronchitis complicated by bronchiectasis.

## 1 CASE REPORT

**Figure 1:**
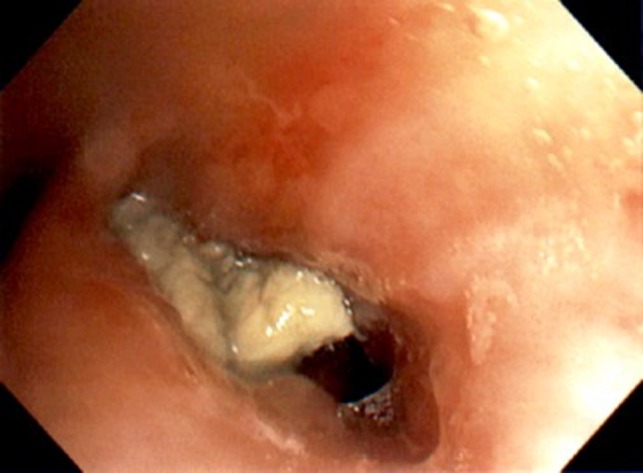
Bronchoscopy: Left lower lobe main bronchus with obstructing thick pale yellow mucus plug with surrounding inflammation

**Figure 2:**
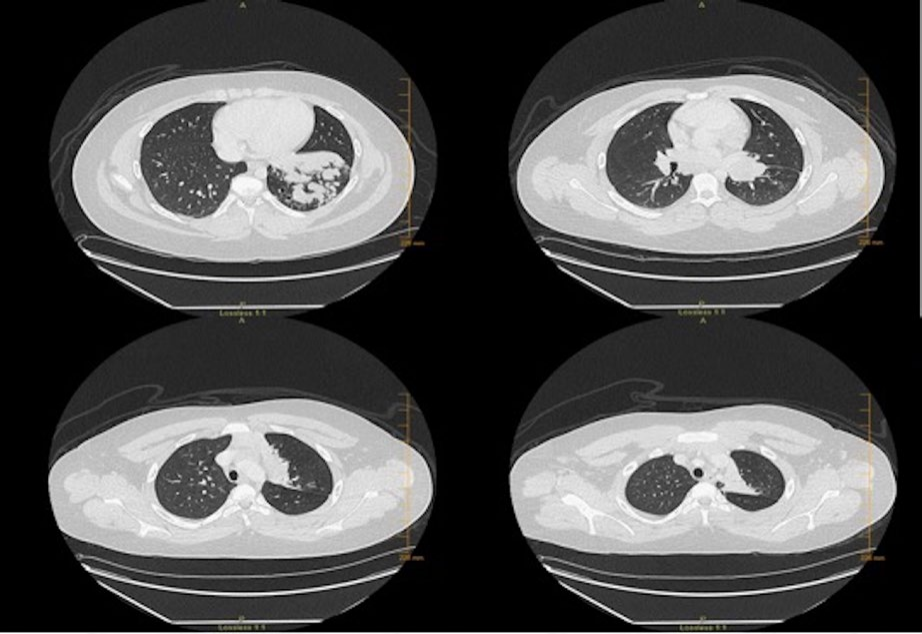
CT Scan: Complete occlusion of left main lower lobe bronchus extending to segmental and sub‐segmental bronchi with partial sparing of superior segmental bronchus, with opacification of diffusely dilated left lower lobe bronchi

A 15‐year‐old African American male presented with a history of persistent pneumonia, dyspnea, expectorating cough with thick yellow sputum, intermittent sharp left‐sided chest pain and single episode of a small amount of hemoptysis. Seven months prior to presentation, he had developed cough and low‐grade fever which was diagnosed as left lower lobe pneumonia and received a course of azithromycin. Two weeks later, he had a relapse of symptoms with worsening of productive cough,yellow‐colored sputum, wheezing, and chest pain which has since continued on and off. There was no history of atopy and no findings to suggest hypersensitivity. No history of travel. Physical examination showed decreased breath sounds bilaterally at the bases with few wet crackles in the left lower chest. Chest X‐ray(CXR) confirmed the presence of persistent left lower lobe nonhomogenous patchy infiltrates, segmental atelectasis, peri‐bronchial inflammation, and concerns of bronchiectasis with adjacent compensatory hyperinflation of lingula and left upper lobe. Pulmonary function test was performed (Table 1). Hematologic evaluation was within normal limits except eosinophilia—20% with absolute eosinophil count of 1.73 × 10^3^/μL. Upon diagnostic fiber‐optic bronchoscopy with bronchoalveolar lavage(BAL): left upper and lower lobe bronchi were obstructed by avascular yellow firm mass although, the lingular bronchus was patent (Figure 1). BAL of left lower lobe showed marked eosinophilia (54%). CT Chest with contrast (Figure 2) was consistent with complete occlusion of left main lower lobe bronchus extending to segmental and subsegmental bronchi with partial sparing of the superior segmental bronchus. Opacification of diffusely dilated left lower lobe bronchi was seen which represents mucoid impaction in the setting of bronchiectasis throughout the basal segments with sparing of superior segments. A similar process was seen involving left upper lobe apical bronchus without associated bronchiectasis. Presence of hilar mass of 2 × 2.6 cm, posterior to left main bronchus was noticed. Based on bronchoscopy results with supportive evidence from CT chest and clinical picture, a diagnosis of plastic/cast bronchitis with eosinophilic lung disease and bronchiectasis was made. Flexible fiber‐optic bronchoscopy & endobronchial ultrasound bronchoscopy were then performed for cast removal with a therapeutic bronchoscope and application of secondary agents. Multiple fine needle aspiration biopsies of the suspicious mass were performed. BAL from right middle lobe showed the presence of eosinophils (26%), left upper lobe eosinophils (8%) & left lower lobe showed eosinophils (54%). Surgical pathology and cytology of the extracted tan friable foreign body of 2.5 × 2.5 × 1.0 cm showed mucoid material with an infiltration of acute inflammation largely of granular eosinophilic cytoplasm with an abundant background of eosinophils. Crystalloid structures compatible with the appearance of Charcot‐Leydon crystals were noticed. The background mucoid material showed light reactivity to mucicarmine and PAS special stain. Consent for lung biopsy was declined by the mother. A summary of work‐up which was concluded as negative (Table 2). Meanwhile, the patient was started on airway clearance therapy with fluticasone, albuterol, mucomyst, 7% hypertonic saline and chest physiotherapy using high‐frequency chest wall oscillation (HFCWO). One‐month course of systemic steroids also failed to improve symptoms, CXR, or eosinophilia. Azithromycin as anti‐inflammatory medication was continued. On follow‐up, the patient reported continued cough with expectoration of thick yellow pellets remaining unchanged over time. Repeat CXR revealed new infiltrate in left upper lobe with worsening of left lower lobe infiltrates due to cast reformation. Flexible bronchoscopy with cryoprobe application was performed with the aim to remove recollected bronchial cast. The left upper and lower lobes with lingular bronchi were observed to be completely obstructed by yellowish thick casts. Persistence and reformation of the unilateral bronchial cast with no clear etiology, in the presence of eosinophilia and bronchiectasis despite comprehensive interventions, leave the case to be managed by periodic cast removal using therapeutic flexible bronchoscopy with suction, cryoprobe, and foreign body extraction basket along with continued airway clearance therapy.

**Table 1:** Pulmonary function test

	Pre	%Ref	Post	%Ref	%Change
VC_(L)_	3.13	80			
FEV_1(L)_	1.99	58	2.26	66	8
FEV_1_/FVC_ratio_	64		72		
FEF_25%__‐__75%__(L/s)_	1.07	27	1.64	42	15
PEF_(L/min)_	6.41	79	6.70	82	3
TLC_(L)_	4.77	94			
DL_co(mL/min/mm Hg)_	21.3	62			

**Table 2:** Summary of work‐up—concluded as negative

Infective	Congenital	Lymphoproliferative immunodeficiency	Autoimmune	Others
TB: Sputum AFB analysis, QuantiFERON Gold	Primary ciliary dyskinesia—electron microscopy exam of the cilia	Flow cytometry from fine needle aspiration biopsy of hilar lymph node	Antibody Panel—SM, SM RNP, SCL 70, Jo 1, SS‐A, SS‐B, ANCA, dsDNA, RF, C3 & C4	Respiratory & food allergy panel
Parasitic infection: stool & BAL ova and parasite	Sickle cell: Hb electrophoresis	Immunoglobulins		Total IgE13kU/L (normal)
BAL fungal culture & serology—Histoplasma, aspergillus, AFB, silver stain—Pneumocystis jiroveci	Alpha1 AT	Complement‐c3, c4, ch50		
	CF: Sweat chloride test, CFTR full gene analysis	T‐ & B‐cell enumeration		

## 2 DISCUSSION

Idiopathic chronic eosinophilic pneumonia (ICEP), a condition extremely rare in the pediatric population, is primarily a pulmonary pathology with eosinophilic infiltration of pulmonary interstitium and alveoli. Symptoms of dyspnea, cough, low‐grade fever, weight loss, and night sweats are observed. CT picture depicts confluent pulmonary consolidation with air bronchogram. Studies focusing on ICEP in children have reported diffuse pulmonary alveolar consolidation with air bronchogram and/or ground‐glass opacity with peripheral predominance, BAL eosinophilia >20% or peripheral eosinophilia >1 × 10^9 ^cells/L, respiratory symptoms for duration >4 weeks in the absence of other known causes of eosinophilic lung disease and consistent open lung biopsy for cases without initial significant clinical and radiological improvement on first line of treatment.^1^ Pulmonary eosinophilia in our patient was found to be complicated by bronchiectasis with the presence of firm bronchial casts, an association of which has never been reported with any eosinophilic lung disease.

Pathophysiology of cast bronchitis may be attributed to injury of alveolar‐capillary barrier, mucosal fragility, elevated venous pressure, lymphatic congestion, or reduced lymphatic drainage. Thus developing, a firm endobronchial cast of rubber‐like or thick gelatinous‐mucoid consistency which adapts to airway architecture causing airway obstruction, infection, and segmental atelectasis. Seear et al classified cast bronchitis based on histological characteristics of the cast into: Type I—eosinophilic or neutrophilic cellularity with a fibrinous or mucinous base with inflammatory respiratory epithelium. The type I cast may be predominantly eosinophilic with Charcot‐Leyden crystals and Curschmann spirals evident upon H&E staining of the lavage fluid. Type II is hypocellular cast consisting of PAS‐positive scant fibrin and mucinous glycoprotein. These have a more chronic presentation with postcardiac procedures indicating injury of lymphatic vessels or imbalanced venous pressure flow.^2^, ^3^, ^4^, ^5^

Management of cast bronchitis can be determined based on its classification. Although the reported literature suggests management of type I cast bronchitis with rigid bronchoscopy using optical forceps or lavage, we were able to manage this case by therapeutic flexible bronchoscope with the use of suction, cryoprobe, and foreign body extraction basket. Use of secondary medications may be employed in association or prior to the extraction procedure, like mucolytic agents which help in breaking up thick tenacious casts. Secondary agents such as, potassium iodide SSKI; urokinase; unfractionated heparin (UFH); fibrinolytic agents (tPA) are used which help in fibrinolysis, whereas N‐acetyl cysteine(NAC) is mucolytic and recombinant DNAase (Dornase alpha) works against cellular DNA, these medications can either be administered by nebulization or applied through bronchoscope intraoperatively. Systemic corticosteroids are employed to reduce secretions; bronchodilators (β_2_ agonists) assist in airway clearance and reducing bronchospasm. Oral macrolides (Azithromycin) reduce inflammation and secretion of cast whereas, hypertonic saline with chest physiotherapy aid in airway clearance.^2^, ^3^, ^4^ Type II casts are removed with bronchoscopy for symptomatic relief, otherwise, it is primarily managed by diagnosing and resolving the underlying etiology.

## CONFLICT OF INTEREST

None declared.

## AUTHOR CONTRIBUTION

HH (Resident Physician, University of Illinois College of Medicine at Peoria): performed as lead author of the manuscript and primary researcher for the case report. NK (Assistant Professor, Department of Clinical Pulmonology, University of Illinois College of Medicine at Peoria): performed as attending physician and supervising author for the case report.
